# Characterization of MicroRNAs Associated with Reproduction in the Brown Planthopper, *Nilaparvata lugens*

**DOI:** 10.3390/ijms23147808

**Published:** 2022-07-15

**Authors:** Ni Wang, Chao Zhang, Min Chen, Zheyi Shi, Ying Zhou, Xiaoxiao Shi, Wenwu Zhou, Zengrong Zhu

**Affiliations:** 1 State Key Laboratory of Rice Biology, Ministry of Agriculture and Rural Affairs Key Laboratory of Molecular Biology of Crop Pathogens and Insects Pests, Key Laboratory of Biology of Crop Pathogens and Insects of Zhejiang Province, Institute of Insect Sciences, Zhejiang University, Hangzhou 310058, China; niwangnw@zju.edu.cn (N.W.); chaozh@zju.edu.cn (C.Z.); chenmin1020@zju.edu.cn (M.C.); shizy0405@zju.edu.cn (Z.S.); shixiao6656@163.com (X.S.); wenwuzhou@zju.edu.cn (W.Z.); 2 Hainan Research Institute, Zhejiang University, Sanya 572000, China; yzhyzb@zju.edu.cn

**Keywords:** microRNAs, *Nilaparvata lugens*, deep sequencing, reproduction, miR-34-5p

## Abstract

Insects have a robust capacity to produce offspring for propagation, and the reproductive events of female insects have been achieved at the molecular and physiological levels via regulatory gene pathways. However, the roles of MicroRNAs (miRNAs) in the reproductive development of the brown planthopper (BPH), *Nilaparvata lugens*, remain largely unexplored. To understand the roles of miRNAs in reproductive development, miRNAs were identified by Solexa sequencing in short-winged (SW) female adults of BPH. Small RNA libraries derived from three developmental phases (1 day, 3 days, and 5 days after emergence) were constructed and sequenced. We identified 905 miRNAs, including 263 known and 642 novel miRNAs. Among them, a total of 43 miRNAs were differentially expressed in the three developmental phases, and 14,568 putative targets for 43 differentially expressed miRNAs (DEMs) were predicted by TargetScan and miRanda. Gene Ontology (GO) and Kyoto Encyclopedia of Genes and Genomes (KEGG) pathway analysis of the predicted miRNA targets illustrated the putative roles for these DEMs in reproduction. The progress events were annotated, including oogenesis, lipid biosynthetic process, and related pathways such as apoptosis, ABC transporters, and amino acid metabolism. Four highly abundant DEMs (miR-9a-5p, miR-34-5p, miR-275-3p, and miR-317-3p) were further screened, and miR-34-5p was confirmed to be involved in the regulation of reproduction. Overexpression of miR-34-5p via injecting its mimics reduced fecundity and decreased *Vg* expression. Moreover, target genes prediction for miR-34-5p showed they might be involved in 20E signaling cascades, apoptosis, and gonadal development, including hormone receptor 4 (HR4), caspase-1 (Cp-1), and spermatogenesis-associated protein 20 (SPATA20). These findings provide a valuable resource for future studies on the role of miRNAs in BPH reproductive development.

## 1. Introduction

Reproduction is fundamental for insects to maintain the population abundance [1]. It has been extensively studied because understanding mechanisms governing reproduction is essential for developing novel strategies for insect control. In most insects, the biosynthesis of vitellogenin (Vg) is a vital process associated with the reproductive success of oviparous species during oogenesis and its accumulation in oocytes [2]. It is often employed as a molecular marker for fecundity and central female reproductive process [3]. In recent years, a significant process has been achieved regarding the mechanisms of reproduction, which includes many factors such as hormone levels, nutrition status, and growth conditions [1]. A network of pathways typically regulates this: the ecdysone hormone pathway [4,5,6], the juvenile hormone pathway [7,8], the insulin pathway [9,10], and the nutritional amino acid/target of rapamycin (AA/TOR) signaling pathway [11,12]. The complex interaction varies greatly depending on the reproductive strategies adopted by insects. Even though many previous studies have revealed much about the essential genes and pathways in regulating insect reproduction, multiple regulatory factors and biological functions in modulating reproduction remain to be announced.

MicroRNAs (miRNAs) are a class of small non-coding (~22 nt) endogenous RNA molecules that extensively regulate gene expression in a variety of ways, including translational repression, mRNA cleavage, and deadenylation [13]. Since the discovery of insect miRNAs in *Drosophila melanogaster*, numerous studies have demonstrated their distinct roles in many critical development events [14,15]. Recent studies have revealed that miRNAs play an essential role in the reproduction of non-Drosophilid female insects such as *Aedes aegypti* [16,17,18,19], *Locusta migratoria* [20,21,22], and *Bombyx mori* [23,24,25]. In addition to the aforementioned, information concerning the potential roles of reproduction-related miRNAs in other insects is limited, especially in agricultural insect pests. Although miRNA profiles in diverse insects have been reported in the past few years, confirmation of their target genes and investigation of their biological significance remains largely unexplored [26,27,28]. Hence, it is essential to decipher insect-specific miRNAs’ biological roles and their contribution to insect reproduction events.

The brown planthopper (BPH), *Nilaparvata lugens*, is a typical monophagous insect herbivore that feeds exclusively on rice—the most important staple food crop globally and a significant food source for about half of the world’s population and has become the most severe and destructive insect pests of rice crops through phloem sap-sucking and nutrient depletion in both temperate and tropical regions of Southeast Asia [29]. This pest can cause severe economic damage during rice production, directly by feeding and indirectly by transmitting several plant viral diseases [30]. The high fecundity based on oogenesis enables planthoppers to flourish populations under appropriate conditions and rapidly develop insecticide resistance under the chemical control [31]. In addition, short-winged (SW) morphs are less mobile than long-winged (LW) morphs but have higher fertility and destructiveness [32]. Therefore, BPH dimorphism greatly exacerbates the extent of agricultural harm and makes this pest challenging to control. Given that safety and feasibility, understanding the molecular basis of pest outbreaks and developing environment-friendly pest-control strategies are crucial to preventing planthopper outbreaks.

Based on the context, we performed a high-throughput Solexa sequencing method to extend the repertoire of SW morphs female adult miRNAs and extensively compare their expression patterns in different developmental phases. We constructed three developmental stages (1 day after emergence, marked 1 d; 3 days after emergence, marked 3 d; 5 days after emergence, marked 5 d) of SW morphs in female adults to find out the miRNAs involved in BPH reproduction. Then, 43 differentially expressed miRNAs (DEMs) were identified, and the targets of these miRNAs were predicted. The miRNAs target prediction and their functional annotations by Gene Ontology (GO) and Kyoto Encyclopedia of Genes and Genomes (KEGG) enrichments were further used to predict the modulatory roles in reproductive development. Four highly abundant differentially miRNAs (*miR-9a-5p*, *miR-34-5p*, *miR-275-3p*, *and miR-317-3p*) associated with reproduction were identified and functionally characterized using miRNA mimics. While the precise targets of these miRNAs need to be investigated further, these discoveries might provide a better understanding of the involvement of miRNAs in BPH reproduction and offer a potential biological foundation for pest management.

## 2. Results

### 2.1. Small RNA Library Construction and Illumina Sequencing

**Figure 1:**
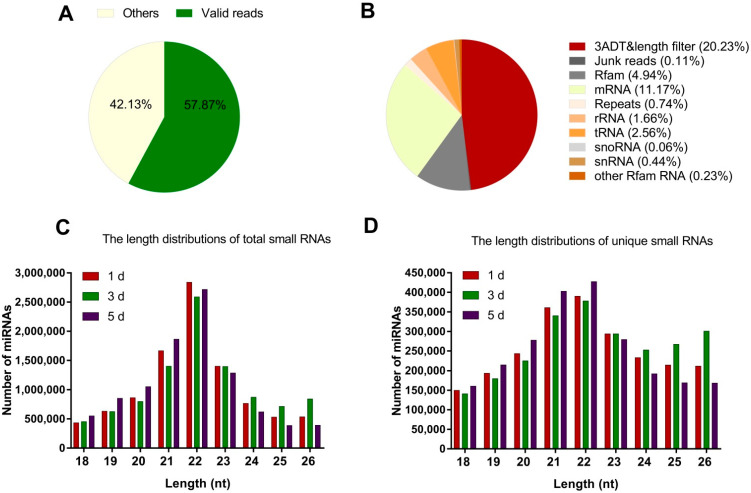
Annotation of small RNAs and length distribution among three small RNAs libraries in *N. lugens*. Annotation of small RNAs (**A**,**B**), total small RNAs reads (**C**), and unique small RNAs reads (**D**). 1 d, 3 d, 5 d represent 1 day after emergence, 3 days after emergence, and 5 days after emergence, respectively. The *x-axis* represents the length of the nucleotide. The *y-axis* represents the number of reads.

To investigate the miRNAs associated with the reproduction of the *N. lugens*, three BPH developmental phases, 1 d (1 day after emergence), 3 d (3 days after emergence), and 5 d (5 days after emergence), were selected to construct three libraries and sequenced by high-throughput Solexa sequencing technology. A total of 15,571,289, 16,362,568, and 15,147,276 raw reads were obtained from three BPH small RNA libraries, respectively (Supplementary Table S1). Of these raw reads, 57.87% were valid miRNAs (Figure 1A), and the remaining 42.13% were other RNAs. Low levels of large fragments, such as mRNA (messenger RNA) and rRNA (ribosomal RNA), were also found, which indicated the high quality and no degradation of RNA samples in the present study (Figure 1B). After removing low quality reads (ambiguous N and ratio >10%, no 3′adapter sequence reads, having 5′adapter contaminations sequence reads, no insert fragment reads, less than 18 nucleotides (nt) reads, containing the poly A/T/G/C reads), we obtained 9,679,096 (61.07%), 9,698,146 (59.03%), 9,737,107 (63.85%) high quality valid reads, which represented 2,290,580 (64.25%), 2,206,291 (61.53%) and 2,292,114 (62.97%) unique reads ranging from 18 to 26 nt (Supplementary Table S1). The length of BPH miRNAs ranged from 18 nt to 26 nt, with 22 nt size comprising 28.29%, 26.38%, and 27.71% of the total reads from three libraries, respectively (Figure 1C). The length of BPH miRNAs ranged from 18 nt to 26 nt, with 22 nt size comprising 16.57%, 15.82%, and 18.54% of the unique reads from three libraries, respectively (Figure 1D).

### 2.2. Identification of Detected miRNAs and Their Expression Profiles

**Figure 2:**
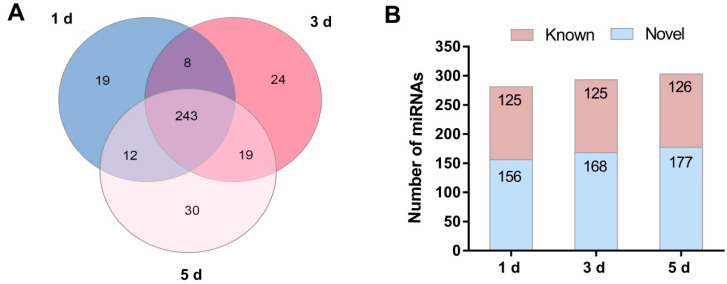
Classification of miRNAs among the three developmental phases in *N. lugens*. (**A**) Venn analysis of all detected miRNAs in 1 d, 3 d, and 5 d, respectively. (**B**) The total number of miRNAs (Known and Novel miRNAs) detected was 1 d, 3 d, and 5 d, respectively. 1 d, 3 d, and 5 d represent 1 day after emergence, 3 days after emergence, and 5 days after emergence, respectively.

Queries of these putative *N. lugens* miRNA reads against the *N. lugens* genomes, and all insect miRNAs in miRBase 22.0 (http://www.mirbase.org/, accessed on 26 January 2022) by BLAST search resulted in the identification of known and novel miRNAs. After removing the redundant sequences, we identified 905 miRNAs, including 263 known and 642 novel miRNAs, given the prefix ‘PC’ (predicted candidate) in the nomenclature we adopted. Specifically, 281, 293, and 303 miRNAs were obtained during the three developmental phases at 1 d, 3 d, and 5 d, respectively. Among all the identified miRNAs (known and novel), 19 miRNAs were detected only in 1 d, 24 miRNAs were found only in 3 d, and 30 miRNAs were only found in 5 d. In comparison, 242 miRNAs were co-expressed during the three developmental phases (Figure 2A). Among these miRNAs, 125 and 156 miRNAs, 125 and 168 miRNAs, and 126 miRNAs and 177 miRNAs were known and novel in 1 d, 3 d, and 5 d, respectively (Figure 2B). The results showed that novel miRNAs accounted for a large proportion and were specific to *N. lugens*. These putatively specific miRNAs were mapped to the *N. lugens* genomes sequences despite having no detectable homology to any known insect pre-miRNAs in miRBase. Then, length distribution was conducted for the identified miRNAs; we found the 22-nt miRNA was the most abundant (25.97%) and showed a distinct unimodal distribution pattern in every library (Supplementary Figure S1). Further, the expression level of the identified miRNAs was analyzed. The results showed that most miRNAs had middle expression levels in the library, whereas a small number of miRNAs had high expression levels (Supplementary Figure S2).

### 2.3. Characterization of Differential miRNA Expression among Three Libraries

**Figure 3:**
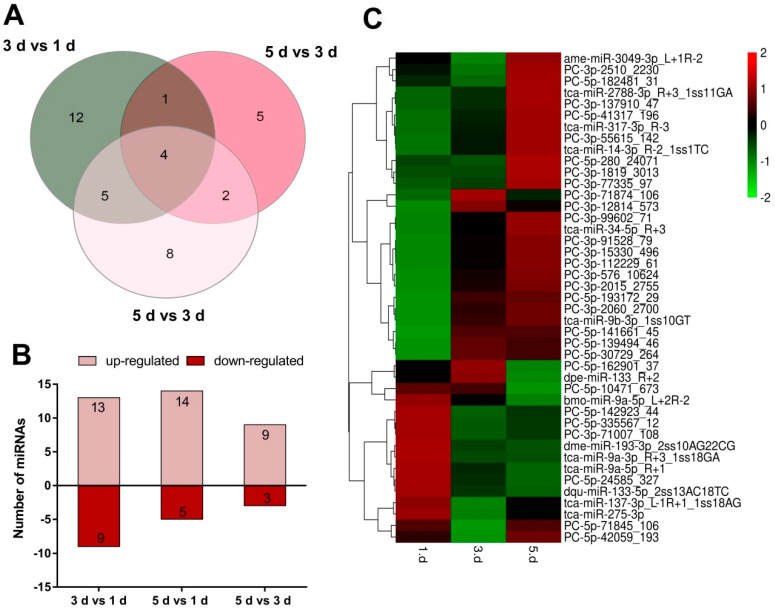
Analysis of differentially expressed miRNAs (DEMs) among the three developmental phases in *N. lugens*. (**A**) Venn analysis of DEMs in 1 d, 3 d, and 5 d. (**B**) Specifically, up- and down-regulation DEMs at each comparison. (**C**) Heatmap of DEMs during the three developmental phases. Red to green represents the up-regulated to down-regulated expression levels. The *x-axis* represents the three developmental phases of BPH, and the *y*-axis represents the differentially expressed miRNAs. 1 d, 3 d, and 5 d represent 1 day after emergence, 3 days after emergence, and 5 days after emergence, respectively.

From all detected miRNAs, a total of 22, 19, and 12 differentially expressed miRNAs were obtained in 3 d compared to1 d (3 d vs. 1 d), 5 d compared to 1 d (5 d vs. 1 d), 5 d compared to 3 d (5 d vs. 3 d), respectively (Figure 3A). Among these differentially expressed miRNAs, 13 and 9 miRNAs, 14 and 5 miRNAs, and 9 and 3 miRNAs were up- and down-regulated at each comparison, respectively (Figure 3B). Based on normalized differences in Illumina read counts, a total of 43 miRNAs were identified as being differentially expressed across three developmental phases of SW morphs female adults; most of them showed middle expression level, and four miRNAs (*miR-317-3p_R-3*, *miR-34-5p_R+3*, *miR-9a-5p_R+1*, *miR-275-3p*) showed high level, three miRNAs (*PC-5p-142923_44*, *PC-5p-182481_31*, *PC-3p-137910_47*) showed low level. The detailed information on the differentially expressed miRNAs (DEMs) is in Supplementary Table S2.

The hierarchical clustering analysis was also performed to analyze the expression profile of 43 DEMs (Figure 3C). These DEMs showed varying frequencies among three libraries. In particular, *PC-3p-71874_106* was significantly highly expressed in the 3 d but lowly expressed in 1 d and 5 d. Some miRNAs revealed relatively low expression in one or two libraries but high expression in another. For instance, *PC-5p-280_24071*, *PC-3p-1819_3013* and *PC-3p-77335_97* showed low expression levels in 1 d and 3 d, but highly expressed in 5 d. In contrast, the sequencing frequency of *PC-5p-142923_44*, *PC-5p-335567_12* and *PC-3p-71007_108* were highly expressed in 1 d. Interestingly, 10 miRNAs (*PC-3p-99602_71*, *miR-34-5p*, *PC-3p-91528_79*, *PC-3p-15330_496*, *PC-3p-112229_61*, *PC-3p-576_10624*, *PC-3p-2015_2755*, *PC-5p-193172_29*, *PC-3p-2060_2700*, *miR-9b-3p*) were significantly lowly expressed in 1 d but highly expressed in the 3 d and 5 d, which consistent with the development of oocyte maturity in the ovary, suggesting its function in ovarian development.

### 2.4. Validation for Differentially Expressed miRNA

**Figure 4:**
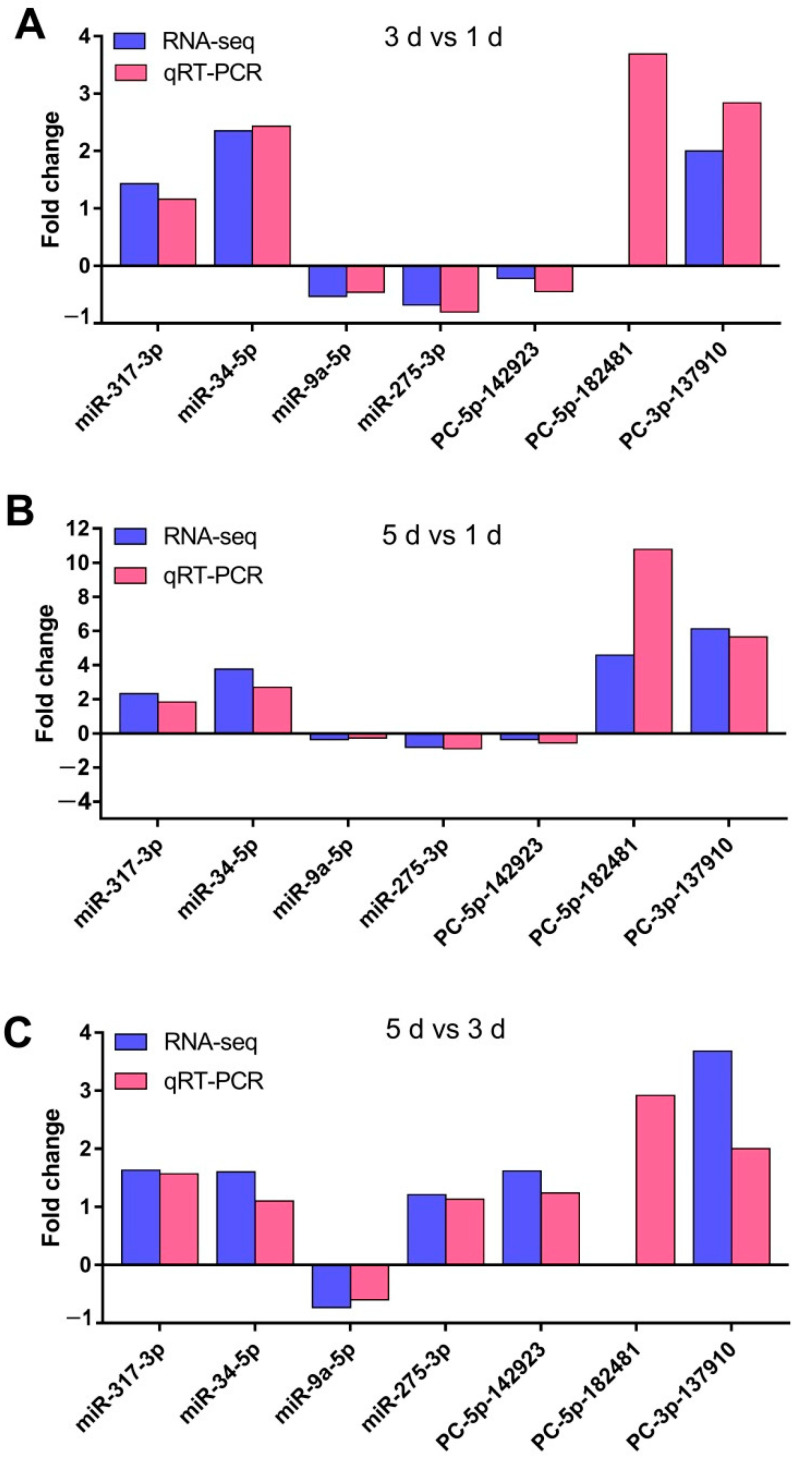
Comparison of RNA-seq results and qRT-PCR analysis of miRNA expression levels. (**A**) FoldChange of seven genes in 3 d vs. 1 d; (**B**) FoldChange of seven genes in 5 d vs. 1 d; (**C**) FoldChange of seven genes in 5 d vs. 3 d; *U6* snRNA was used as an internal reference for the qRT-PCR. The *x*- and *y*-axis represent the miRNAs and FoldChange levels, respectively. 3 d vs. 1 d represents 3 d compared to 1 d, 5 d vs. 1 d represents 5 d compared to 1 d, and 5 d vs. 3 d represents 5 d compared to 3 d, respectively. 1 d, 3 d, and 5 d represent 1 day after emergence, 3 days after emergence, and 5 days after emergence, respectively.

To validate the transcript abundance of the identified miRNAs in the sRNA sequencing, four highly abundant DEMs (*miR-9a-5p*, *miR-34-5p*, *miR-275-3p*, *and miR-317-3p*) and three low abundance expression DEMs (*PC-5p-142923_44*, *PC-5p-182481_31*, *PC-3p-137910_47*) were selected from three developmental stages for validating their relative expressions by quantitative real-time polymerase chain reaction (qRT-PCR) using the same total RNA samples employed for the construction of the sequencing libraries (Figure 4). The qRT-PCR results showed that their expression patterns were similar to those of the high-throughput sequencing. Although the degree of relative fold changes of the DEMs obtained via sRNA sequencing and qRT-PCR analysis was different, the trend was the same.

### 2.5. Prediction and Annotation of Differentially Expressed miRNA Target Genes

**Figure 5:**
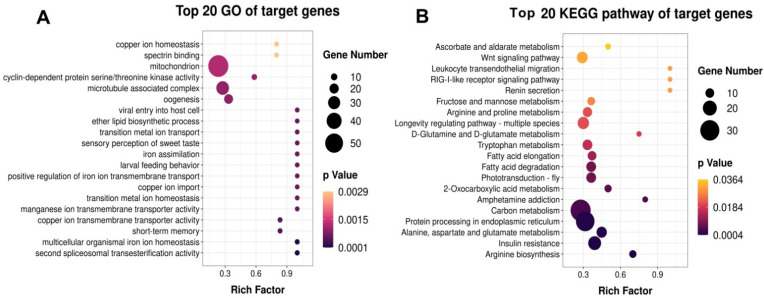
Gene Ontology (GO) and Kyoto Encyclopedia of Genes and Genomes (KEGG) enrichment analysis of differentially expressed miRNAs in *N. lugens*. (**A**) GO enrichment analysis of the putatively targeted genes of differentially expressed miRNAs. (**B**) KEGG enrichment analysis of the putatively targeted genes of differentially expressed miRNAs. Different colors show the Q-values, and less than 0.05 denotes a significant enrichment. Different sizes of dots mean the gene number enriched in a term or pathway.

To identify the specific functions of 43 DEMs, putative targets for these miRNAs within the 3′UTRs of transcripts from the *N. lugens* were predicted using TargetScan and miRanda. A total of 14,568 putative targets were obtained (Supplementary Table S3). The annotation of the target genes of DEMs was performed using Gene Ontology (GO) enrichment to functionally characterize the miRNA targets. The results showed that 43 DEM target genes’ GO function was mainly enriched in the mitochondrion, microtube-associated complex, oogenesis, cyclin-dependent protein serine/threonine kinase activity, and lipid biosynthetic process (Figure 5A). Subsequently, we found that these putative targets were significantly (*p* ≤ 0.05) categorized into 144 terms (Supplementary Table S4). Among these terms, 89 GO terms of biological processes were identified, and genes involved in reproductive development processes (e.g., GO: 0048477: oogenesis with 57 targets; GO: 0007560: imaginal disc morphogenesis with three targets; GO: 0016055: Wnt signaling pathway with 31 target genes; GO: 0048190: wing disc dorsal/ventral pattern formation with 15 target genes; GO: 0046329: negative regulation of JNK cascade with nine targets. GO: 0048599: oocyte development with two targets) were found. In the molecular function category, 33 GO terms were identified, including transmembrane transporter activity (GO: 0022857), cyclin-dependent protein serine/threonine kinase activity (GO: 0004693), and actin filament binding (GO: 0051015). Moreover, we also found that 22 GO terms were significant in the cellular component category, such as an integral component of membrane (GO: 0016021) with 331 targets, cytosol (GO: 0005829) with 252 targets, mitochondrion (GO: 0005739) with 248 targets.

Following GO analysis, the target genes were also uploaded into the Kyoto Encyclopedia of Genes and Genomes (KEGG) database to identify the pathways actively regulated by miRNA in *N. lugens*. KEGG pathway enrichment analysis predicted that putative target genes were significantly (*p* ≤ 0.05) enriched in 20 pathways (Figure 5B and Supplementary Table S5), including Insulin resistance (KO04931, *p*-value = 0.0004), Wnt signaling pathway (KO04310, *p*-value = 0.0317), D-Glutamine and D-glutamate metabolism (KO00471, *p*-value = 0.0188), Longevity regulating pathway (KO04213, *p*-value = 0.0188). Additionally, Endocytosis (n = 105), FoxO signaling pathway (n = 37), Notch signaling pathway (n = 20), Hippo signaling pathway (n = 41), TGF-beta signaling pathway (n = 21), mTOR signaling pathway (n = 24), MAPK signaling pathway (n = 22), Insulin signaling pathway (n = 13), apoptosis (n = 33) and ABC transporters (n = 20) were predicted to be involved in other important developmental processes (Supplementary Figure S3). It is speculated that DEMs may modulate important signaling pathways by regulating the expression of putative target genes, thus affecting the reproductive development in *N. lugens*.

### 2.6. Functional Analysis of miR-34-5p In Vivo

**Figure 6:**
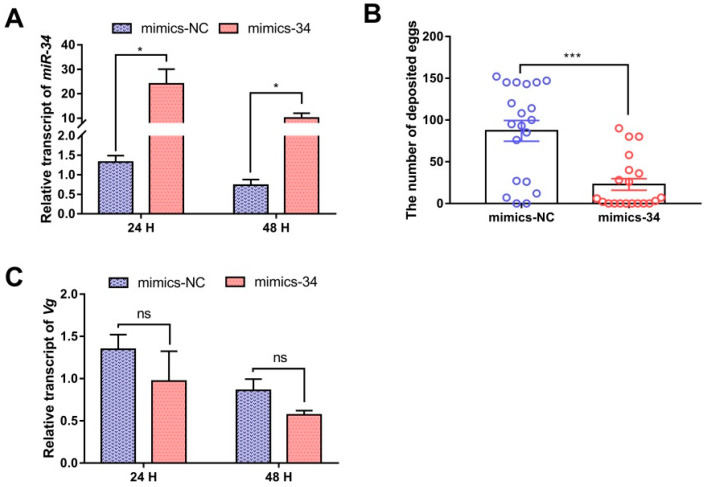
Functional analysis of *miR-34-5p* in vivo. (**A**) Determination of *miR-34-5p* abundance expression after injection of mimics-34. (**B**) Effect of fecundity after injection of mimics-34. (**C**) Relative expression of *Vg* after injection of mimics-34. Data are expressed as the mean ± SEM (n ≥ 3), and the differences between the two groups were analyzed using a two-tailed unpaired student *t*-test. Results were considered statistically significant if the *p*-value was < 0.05. (* *p* < 0.05, *** *p* < 0.001, ns means no significant difference).

Four highly abundant DEMs (miR-9a-5p, miR-34-5p, miR-275-3p, and miR-317-3p) were screened to validate the function of these differentially expressed miRNAs. We consider the possibility that four miRNAs may influence the fecundity of *N. lugens*. To further understand the functional roles of four miRNAs in reproduction, we investigated ovulation experiments following miRNA mimics injected in newly emerged adult females. Compared to the control group injected with mimics-NC, the abundance of *miR-34-5p* increased by 25- and 15-fold 24 and 48 h post-injection, respectively (Figure 6A). Following mating with non-injected males, females treated with mimics-34 had fewer offspring, with an average of 87.00 per female during the first seven days, a 22.80% decrease compared with the number of offspring produced by mimics-NC-injected controls (Figure 6B). However, the females injected with *miR-9a-5p*, *miR-275-3p*, and *miR-317-3p* did not lay significantly fewer eggs than control females (Supplementary Figure S4), suggesting that *miR-34-5p* is required for female reproduction. *Vg* is the critical factor in vitellogenesis in insects. We, therefore, investigated the effects of mimics-34 on expression levels of *Vg* transcription in the female adult whole body. The qRT-PCR analysis indicated that the relative transcript of *Vg* in the mimics-34-injected group decreased by 28% and 34% 24 and 48 h post-injection, respectively (Figure 6C). These indicated that *miR-34-5p* might act as an influential regulatory factor involved in the reproductive development of *N. lugens*.

### 2.7. GO and KEGG Annotation of miR-34-5p Target Genes

**Figure 7:**
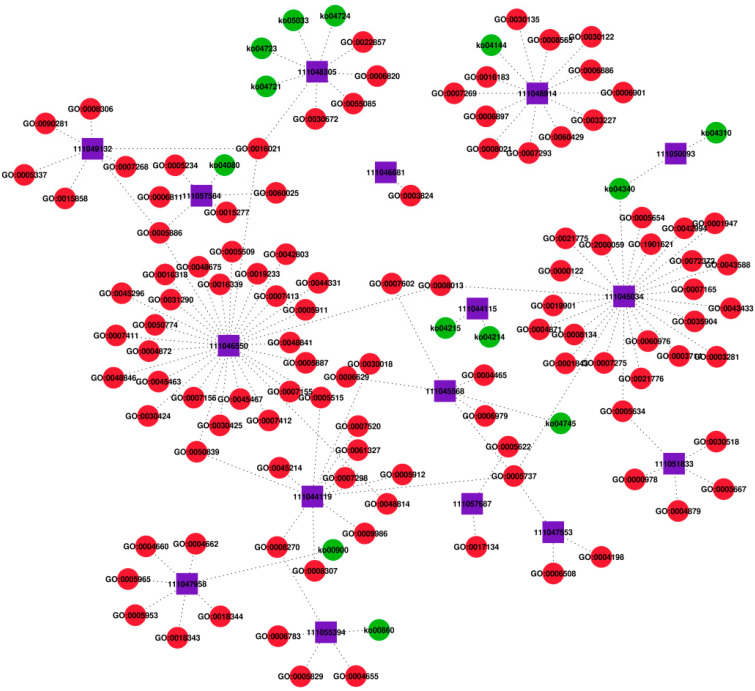
GO-targets-KEGG network of *miR-34-5p*. The purple box represents Gene ID for putative targets of *miR-34-5p*. The red circle represents GO terms for putative targets of *miR-34-5p*. The green circle represents the KEGG pathway for putative targets of *miR-34-5p*. The line represents the interaction between target genes.

To preliminary explore the mechanism of *miR-34-5p* in reproduction, the target genes of *miR-34-5p* with GO and KEGG annotations were searched and analyzed, and the GO-targets-KEGG network was developed. The detailed information on predicted results is listed in Supplementary Table S6. The results showed that the targets of *miR-34-5p* were enriched in multiple physiological functions. One target gene may correspond to various physiological functions, and several target genes may correspond to the same physiological function (Figure 7). The *Hormone receptor 4* (*HR4*) (Gene ID: 111051833), which acts as a transcriptional repressor in the 20E signaling cascades, was targeted by *miR-34-5p*, and the intracellular steroid hormone receptor signaling pathway (GO: 0030518) was found. Therefore, we speculated that *miR-34-5p* mediated insect hormones to modulate reproductive development. In addition, the *Spermatogenesis-associated protein 20* (*SPATA20*) (Gene ID: 111046811), the gonadal development-related gene, was also obtained and mainly annotated to GO term related to the catalytic activity (GO: 0003824), indicating that *miR-34-5p* may participate in reproduction via the regulation of protein activity. Moreover, the *Caspase-1-like* (Gene ID: 111044115), which encodes an effector caspase involved in the execution of apoptosis, was also identified as a target linked with apoptosis (KO04214; KO04215). This result suggested that *miR-34-5p* may regulate insect reproduction by activating apoptosis. Our finding lays a fundament for further studying the mechanism of reproductive regulation of *N. lugens*.

## 3. Discussion

High reproductive capacity is an essential strategy insects use to increase their chances of survival in nature and their ability in various ecological environments. Molecular studies have revealed much about the relationships between genes and pathways concerning the regulation of insect reproduction. However, the precise roles of miRNAs in insect reproduction remain unresolved. Here, we focused on identifying miRNAs regulating reproduction at different developmental phases of SW morphs in female adults. Adult libraries may be able to provide certain reproduction-related information. First, a total of 9.68 million, 9.70 million, and 9.74 million high-quality valid reads were determined through deep sequencing of 18 to 26 nt in the three BPH small RNA libraries (1 d, 3 d, and 5 d), which is similar to the number of miRNAs reported in a previous study of BPH [33]. The length distributions of the total small RNA reads and unique small RNA reads showed a distinct unimodal distribution pattern in every library with peaks at 22 nt. The 22 nt reads were shown to be proven miRNAs as was found previously [27,34] and are consistent with the size standard for miRNAs resulting from the abundant dicer-derived products as well as analogous for the length distribution observed in *D*. *melanogaster* [35] and *Acyrthosiphon pisum* [36]. However, a previous study showed BPH existed in a bimodal distribution of total small RNA reads, one peak at 21–22 nt and another at 27–28 nt [33,37,38]. One possible explanation is that in our study, only the total small RNA reads and unique small RNA reads of 18 to 26 nt in length were included in the length distribution analysis. Hence the results were slightly different. Furthermore, the findings revealed that miRNA molecules vary in length, possibly due to asymmetric structural motifs in various precursors [39].

By sequencing small RNA libraries, we discovered 905 miRNAs, including 263 conserved and 642 novel miRNAs. The results showed that many miRNAs are novel, indicating that these specific miRNAs may regulate reproductive development in BPH. It is necessary to note that we only analyzed the small RNAs in three developmental phases, additional novel and useful small RNAs could be detected in more different developmental stages. The frequency of short nucleotide reads generally are highly representative of relative abundance and was used to estimate the expression level of miRNAs [40]. Highly expressed miRNAs would likely have a large number of sequenced reads and provide some essential insight into the function of these miRNAs [41].

Nevertheless, our findings suggest that most miRNAs were middle abundant, while the number of highly expressed miRNAs was relatively small. The results revealed that specific miRNAs might be expressed in particular phases or limited physiological processes at moderate levels. Differences in miRNA abundance may be connected to the roles that miRNAs may play in insect development, implying a potential role in the temporal or spatial suppression of specific target mRNAs.

We identified 43 differentially expressed miRNAs across three libraries, including 29 novel and 14 conserved miRNAs. These differentially expressed miRNAs (DEMs) imply that miRNAs are associated with the BPH reproductive development and can regulate several critical processes via multiple pathways and targets. However, many miRNAs are novel, and their biological functions remain unclear. Thus, more research is needed to elucidate the roles of these miRNAs. GO enrichment suggested possible involvement in various biological processes, cellular components, and molecular functions of putative target genes of 43 DEMs (Supplementary Figure S4). The predicted target genes were mostly annotated to 50 GO terms related to the oxidation-reduction process, cytoplasm, and ATP binding across three developmental phases. It is well documented that the quality of mature oocytes is not only associated with the maturation of the nucleus (meiosis) but also closely linked to cytoplasmic changes [42], and the maturation of oocytes is well coordinated with the mitochondria redistribution [43].

Furthermore, mitochondrial failure enhanced ROS generation, whereas excessive ROS caused oxidative stress, which hampered the maturation of oocytes [44]. The GO enrichment results indicate that the target genes of these DEMs may play a crucial role in oocyte development by regulating various physiological activities. Besides, we also observed that the GO function of target genes of 43 DEMs were primarily enriched in the mitochondrion, microtube-associated complex, oogenesis, cyclin-dependent protein serine/threonine kinase activity, lipid biosynthetic process, etc. (Figure 5A). Mitochondria are the energy factories of oocytes and one of the indicators to measure the quality of oocytes, which play a vital role in cell metabolism, growth, and proliferation [45]. Regarding the lipid biosynthetic process, sphingolipids are an important class of lipids that participate in crucial events of cell proliferation, differentiation, and apoptosis in various species [46]. Recently, evidence has accumulated to show that sphingolipids have roles in the signaling pathways involved in reproduction and oocyte development of *N. lugens* [47,48,49]. Cyclin-dependent kinase (CDK) is a serine/threonine protein kinase family that cooperates with cyclin and is required for embryo development and cell cycle progression [50]. Taken together, we speculated that the target genes of DEMs might be involved in the reproductive development of *N. lugens*.

Following that, KEGG analysis showed that the top 20 pathways were mainly involved in Insulin resistance, Wnt signaling pathway, and amino acid metabolism such as glutamate metabolism, etc. (Figure 5B), implying their critical roles in the regulation of reproduction and development in *N. lugens*. Among these, glutamine, an essential amino acid involved in metabolism in organisms, activates the TOR pathway to regulate the fecundity of *N. lugens* by mediating *Vg* expression [51]. Some pathways, such as endocytosis, apoptosis, ABC transporters, etc., were also discovered. ABC transporters are primary-active proteins that mediated the transport of many substrates, including across membranes, including nutrients and lipids [52,53], and was reported to be required for cuticle barrier formation in *L. migratoria* [52]. Endocytic systems serve many critical cellular functions, including nutrient absorption, cell-surface receptor expression modulation, cell polarity maintenance, and antigen presentation. Vitellogenin receptors (VgRs) mediate Vg endocytosis in oviparous insects, which is critical for reproduction [54]. These results suggest that DEM targets may play essential roles in energy allocation and nutrient transport.

Interestingly, four highly abundant DEMs (*miR-9a-5p*, *miR-34-5p*, *miR-275-3p* and *miR-317-3p*) were identified in our study. The *miR-317* has been linked to the spatiotemporal regulation of *cyclin B* during *Drosophila* early embryogenesis [55]. Furthermore, *miR-317* has been reported to participate in the larval ovary morphogenesis of *Drosophila* [56]. The functions of *miR-100*/*miR-317* and *miR-100*/*miR-285* are explored in larva and pupa, respectively, implying the regulatory role of *miR-317* in the developmental transitions of *Bactrocera dorsalis* [57]. In the present study, *miR-317* was lowly expressed at 1 d and 3 d but highly expressed in 5 d of BPH, suggesting that *miR-317* is a critical regulator of oocyte maturation. Previous studies demonstrated that *miR-275* is indispensable for blood digestion and egg development in the mosquito *A. aegypti* [16]. Recently, ecdysone receptor regulates *miR-275* to facilitate egg development [58]. Besides, *miR-275* targets *Vg-2* and regulates the function of blood digestion and ovary development [59]. These findings will improve the molecular understanding of ovary development and reproduction. We found that *miR-275* was highly expressed on the first day, indicating its role in early ovarian development. Several conserved miRNAs have been shown to regulate wing development, including *miR-34* and *miR-9b*, which have been confirmed to modulate the wing polymorphism and wing formation. In *Drosophila*, the mutants of *miR-9* repressed apoptosis during wing development, resulting in the deletion of wing margins [60]. In *N. lugens*, *miR-9b* was reported to regulate wing length in BPHs between insulin receptor genes and *NlUbx* via a regulatory cascade [61]. In addition, *miR-9b* mediating wing dimorphism and development was confirmed in the brown citrus aphid *Aphis.* *citricidus* based on the interaction with the insulin and insulin-like signaling pathway [62]. In this study, we focused on the impact of miRNA on ovarian reproductive development. Thus we did not explore its role in wing development; other functions will need to be investigated in the future. In *B*. *mori*, *miR-34* modulates larval growth and wing morphogenesis by directly modulating ecdysone signaling and cuticle protein [25]. Besides, *miR-34* modulates a positive autoregulatory feedback loop of JH and insulin/IGF signaling (IIS) pathway to control wing polyphenism in BPH [63]. In our current study, the expression of *miR-9* and *miR-34* demonstrated a similar expression pattern, they are highly expressed in 3 d and 5 d but lowly expressed in 1 d. The expression patterns implied that *miR-34* and *miR-9* might be implicated in BPH reproductive development.

Based on the above, we conducted egg-laying experiments, but no significant effect of *miR-9a-5p*, *miR-275-3p*, and *miR-317-3p* was observed, except *miR-34-5p*. We suspected that they might play a minor role in reproductive development, and other functions need to be explored. We found that overexpression of *miR-34-5p* via its mimics reduced fecundity and decreased *Vg* expression. The target genes of *miR-34-5p* with GO and KEGG annotations were searched and evaluated, and the GO-targets-KEGG network was constructed. *Caspase-1*, *hormone receptor 4* (*HR4*), and *spermatogenesis-associated protein 20* (*SPATA* *20*) were predicted that they might be one of its target genes. Previous studies showed *HR4* is required for successful vitellogenesis and oogenesis during the adult stage [64], and injection of ds*HR4* (double-stranded) in the final instar nymphs arrested development [65]. We concluded that *miR-34-5p* might influence the reproduction of *N. lugens* by mediating the 20E signaling pathway. The SPATA (Spermatogenesis-associated gene family) is involved in spermatogenesis, sperm maturation, and fertilization. For example, *SPATA 20* was identified as one of the testicular genes altered in obese mice [66], and it was found to be involved in response to DNA damage in sperm, thereby affecting sperm quality [67]. Another study implicated that reduced expression of *SPATA 5* led to decreased *Vg* expression and fecundity of *N. lugens* [68]. We hypothesized that *miR-34-5p* might affect the fecundity by targeting *SPATA 20* via mating behavior. Growing evidence suggests that caspase is a transcriptional regulator of cell death by modulating the components of apoptosis. It is essential for oocyte development and normal larval development in *B. dorsalis* [69]. Further evidence is needed to clarify the functions of *miR-34-5p* in regulating reproduction.

MiRNAs play crucial roles in the regulation of multiple biological processes in insects. Thus, we speculated the possibility that the artificial *miR-34-5p* has excellent potential to be developed as a novel next-generation insecticide. The future objective is to move research findings from the laboratory to the rice field to keep the environment healthy and productive. However, the persistence and penetrability of *miR-34-5p* should be investigated in field conditions further, and insect toxicological effects also need to be tested. Molecular biology methods are widely used for sustainable pest management as environment-friendly pest-control strategies due to safety and feasibility. Successful overexpression or knocking down of miRNA through injection or feeding with artificial diets led to strong and long-lasting phenotypic effects, i.e., increased lethality, low fecundity, developmental defects, and altered feeding behavior. These phenotypes will help to reduce the pest population and inhibit its development to some extent. Achieving effective pest management by inhibiting specific pest genes and protecting non-target organisms is an attractive option for governance by exploiting important environmental intrinsic factors and targets. In addition, promoting biological control strategies that combine natural enemies with other non-chemical approaches and plant resistance will also help reduce pesticide use and the risk of planthopper outbreaks. These discoveries provide a solid foundation for developing miRNA-based green pest control technology to help mine the key factors in insect reproduction.

## 4. Materials and Methods

### 4.1. Sample Collection and Total RNA Extraction

This study constructed three short-winged (SW) morphs of female adults with small RNA libraries for three developmental phases (1 day after emergence, marked 1 d; 3 days after emergence, marked 3 d; 5 days after emergence, marked 5 d). The samples of *N. lugens* each were composed of 30 insects with a similar scale. All the samples in this study originated from a field population in the Huajiachi campus of Zhejiang University, Hangzhou, China. The BPHs were reared on susceptible rice seedlings cv. Taichung Native 1 (TN1) at 27 ± 1 °C, 70% relative humidity, and a 16:8 h light: dark photoperiod as described in a previous study [47]. All samples were frozen in liquid nitrogen immediately and stored at −80 °C. Total RNA was extracted using TRIzol Reagent (Invitrogen, Carlsbad, CA, USA) according to the manufacturer’s protocol. The quantity of the total RNA was accessed by NanoDrop™ 2000 spectrophotometer (Thermo Fisher Scientific, Waltham, MA, USA), and the integrity of the RNA was measured using the Agilent 2100 Bioanalyzer (Agilent Technologies, Palo Alto, CA, USA). Only A260/A280 ratio lies between 1.8 and 2.0, and an RNA integrity number > 7.0 can be accepted.

### 4.2. Small RNA Library Construction and Deep Sequencing

For small RNA library construction, total RNA extraction from the *N. lugens* of SW morphs female adults for three developmental phases were pooled and prepared according to the TruSeq Small RNA Sample Preparation Kits (Illumina, San Diego, CA, USA). In brief, Solexa sequencing was performed as follows. For each library, small RNA fragments measuring 18–26 nt were collected using PAGE gel, then ligated to 3′ chimeric oligonucleotide adaptors (5′-TGGAATTCTCGGGTGCCAAGG-3′) and 5′ (5′-GTTCAGAGTTCTACAGTCCGACGATC-3′) to its end of the RNA pool using T4 RNA ligase (Illumina, San Diego, CA, USA). The adaptor-ligated small RNAs then were reverse transcribed and used as templates for cDNA synthesis using Superscript II reverse transcriptase (Invitrogen, Carlsbad, CA, USA). The cDNAs were amplified using Illumina’s small RNA primer sets with 15 PCR cycles needed to produce the sequencing libraries. The purified PCR amplification products were sequenced on an Illumina HiSeq 2500 at LC-Bio Tech (Hangzhou, China) according to the manufacturer’s protocol. Single-end read of 50-bp length was obtained.

### 4.3. Sequence Analysis and miRNAs Annotation

The raw reads were processed into clean reads using an in-house program, ACGT101-miR (LC Sciences, Houston, TX, USA). First, raw reads were filtered to remove low-quality reads (ambiguous N and ratio >10%, no 3′adapter sequence reads, having 5′adapter contaminations sequence reads, no insert fragment reads, less than 18 nt reads, containing the poly A/T/G/C reads). The remaining reads were analyzed by Rfam (http://rfam.xfam.org, accessed on 26 January 2022) and Repbase (http://www.girinst.org/education/index.html, accessed on 26 January 2022) to discard messenger RNA (mRNA) ribosomal RNA (rRNA), transfer RNA (tRNA), small nuclear ribonucleic acid (snRNA), small nucleolar RNA (snoRNA) and repeat sequences. Based on the analysis and statistics of the raw data, we further calculated the length distribution of the total number and a unique number of the filtered valid data.

Subsequently, unique sequences with lengths in 18~26 nt were mapped to BPH precursors in miRBase 22.0 (http://www.mirbase.org/, accessed on 26 January 2022) by BLAST search to identify known miRNAs (conserved miRNA) and novel 3p- (from the 3′ arm of predicted miRNA precursor) or 5p- (from the 5′ arm of predicted miRNA precursor) derived miRNAs. Length variation at both 3′ and 5′ ends and one mismatch inside the sequence were allowed in the alignment. The unique sequences mapping to the other arm of known insect precursor hairpin opposite the annotated mature miRNA-containing arm were considered novel 5p- or 3p derived miRNA candidates.

The remaining sequences were mapped to other insect species precursors in miRBase 22.0 by BLAST search. The mapped pre-miRNAs were further BLASTed against the BPH genomes to determine their genomic locations. The aforementioned miRNAs were considered known miRNAs. The unmapped sequences were BLASTed against the BPH genomes, and the hairpin RNA structures containing sequences were predicted from the flank 80 nt sequences using RNA fold software (http://rna.tbi.univie.ac.at/cgi-bin/RNAfold.cgi, accessed on 26 January 2022). The criteria for miRNA annotation and secondary structure formation are listed in Supplementary Table S7. Data normalization followed the procedures as described in a previous study with minor modifications (Supplementary Table S8).

### 4.4. Analysis of Differential miRNAs Expression

MicroRNAs were regarded as differentially expressed based on normalized deep-sequencing levels (with the exclusion of 10 RPM) in development. The *p*-value was estimated selectively using an ANOVA (http://en.wikipedia.org/wiki/One-way_analysis_of_variance, accessed on 26 January 2022) (multiple groups) or Student’s *t*-test (http://en.wikipedia.org/wiki/Student’s_t-test, accessed on 26 January 2022) (two groups). A significance threshold level was set to be 0.05 in each test. miRNAs with *p*-value < 0.05 and log2 (fold change ratio) > 1 were considered as differentially expressed miRNAs (DEMs).

### 4.5. Quantitative Real-Time PCR (qRT-PCR) Analysis

The first-strand cDNA of mature miRNAs and mRNA was performed using ReverTra Ace qPCR RT Master Mix (Stem-loop method) (Toyobo Co., Osaka, Japan) and ReverTra Ace qPCR RT Master Mix with gDNA Remover (Toyobo Co., Osaka, Japan), respectively. For the miRNA, each 10-μL reverse transcription reaction system contained 1 μg total RNA, 2 μL 5 × RT Buffer, 0.5 μL RT Enzyme Mix, 0.5 μL miRNA-specific stem-loop RT primers (49 nt, the frontal 36 nt were the stem-loop region, they were stationary; the last 13 nt were reverse complementary to the 3′ portion of the miRNA molecules), and the reactions were incubated in S1000 Thermal cycler PCR machine (Bio-Rad) for 37 °C 15 min, 98 °C 5 min and then held at 4 °C. For the mRNA, 1 μg of total RNA was reverse-transcribed to cDNA in a 10-μL reaction, according to the manufacturer’s recommendations. The primers for the qRT-PCR of miRNAs all contain forward primer (ACACTCCAGCTGGGT, 15 nt of 5′ portion were stationary and 13–15 nt were the 5′ portion sequence of the miRNAs) and universal reverse primer (CTCAACTGGTGTCGTGGAGTCGGCAA).

Expression levels of *miR-34-5p* and *Vg* were determined by qRT-PCR using an ABI 7300 Real-Time PCR System (Applied Biosystems, Branchburg, NJ, USA) with standard SYBR Green Real-time PCR Master Mix (Toyobo Co., Osaka, Japan). In each reaction, 20 μL reaction mixtures containing 1 μL cDNA (five-fold dilution) were prepared and incubated at 95 °C for 1 min, 40 cycles of 95 °C for 10 s and 60 °C for 30 s, followed by the melting curve analysis (68 °C–95 °C) to confirm the specific PCR amplification products. Three biological replicates with three technical replicates were conducted for each qRT-PCR. The expression levels of miRNA were normalized to U6 snRNA (small nuclear RNA), and the expression levels of *Vg* were normalized to *β*-actin. The relative expression level of miRNA and mRNA were calculated according to the arithmetic formula 2^−ΔΔ*ct*^ [70]. The significance of differential expression of each candidate miRNA was assessed using a two-sample *t*-test. Results were considered statistically significant if the *p*-value was <0.05. The primers used for the qRT-PCR are listed in Supplementary Table S9.

### 4.6. Prediction and Annotation of miRNAs Target Genes

To predict the genes targeted by differentially expressed miRNAs, two computational target prediction algorithms (TargetScan 50, http://www.targetsacn.org, accessed on 1 March 2022, using its default parameters and miRanda3.3a, http://www.miRNA.org, accessed on 1 March 2022, Max Energy < −10) were used to identify *N.* *lugens* miRNA binding sites. The sequences of the 3′UTRs of the target genes in *N. lugens* were downloaded from the National Center for Biotechnology Information (NCBI) (http://www.ncbi.nlm.nih.gov/, accessed on 1 March 2022). Finally, the data predicted by both algorithms were combined, and the overlaps were calculated. Enrichment analysis of the predicted target genes was conducted with Gene Ontology (GO) (http://www.geneontology.org/, accessed on 1 March 2022) and the Kyoto Encyclopedia of Genes and Genomes (KEGG) pathway (http://www.genome.jp/kegg/, accessed on 1 March 2022). Terms and pathways with a *p* < 0.05 were considered to be significantly enriched.

### 4.7. Characterization of miRNA Candidates on Reproductive Development

To explore the function in vivo on *N. lugens* reproductive development for the four highly abundant differentially miRNAs, the miRNA mimics and the negative control mimics (mimics-NC) that did not target any gene in *N. lugens* were designed and synthesized by Genepharma (Shanghai, China) (Supplementary Table S10). The mimics were injected into the side position between the middle leg and hind leg of BPH. Each BPH was injected with 50 nL of miRNA mimics (20 μM) using a FemtoJet microinjector (Eppendorf, Hamburg, Germany). Then the BPHs were treated with mimics reared on rice plants for further experiment.

In the fecundity bioassays, each newly emerged SW female adult was used. The treatment group (injected with miRNA-mimics) and control group (injected with mimics-NC) each included 20 pairs of adults. A female and two untreated male adults were separately reared on 1-month-old TN1 fresh rice plants in glass tubes (45 cm in depth, 5.5 cm in diameter) for 7 days. After 7 days, the pairs from the tubes were removed after a successful mating for another 7-day-oviposition observation. The number of hatched offspring laid by the female adults was recorded during the following seven consecutive days. After that, rice seedlings were dissected, and the number of eggs on them that failed to hatch was also counted.

## Data Availability

Small RNA sequencing data have been deposited to National Center for Biotechnology Information (NCBI) Gene Expression Omnibus (GEO) database (Accession Number: GSE174315). All other relevant data are included in the main text and supporting information.

## Supplementary Materials

Supplementary materials are uploaded at https://www.mdpi.com/article/10.3390/ijms23147808/s1.
