# Supplementary material for: Characterization of MicroRNAs Associated with Reproduction in the Brown Planthopper, Nilaparvata lugens
Source: Int J Mol Sci. 2022 Jul 15;23(14):7808. doi: 10.3390/ijms23147808 (PMC9316625; doi:10.3390/ijms23147808)
Supplement: Supplementary file 1 [file ijms-23-07808-s001.zip › Supplementary materials.pdf]

Supplementary Materials: Characterization of MicroRNAs Associated with Reproduction in the Brown Planthopper, *Nilaparvata lugens*

Ni Wang, Chao Zhang, Min Chen, Zheyi Shi, Yin Zhou, Xiaoxiao Shi, Wenwu Zhou and Zeng-Rong Zhu

Supplementary Materials:

Figure S1. Length distribution of identified small RNAs in the three developmental phases in *N. lugens*.

Figure S2. Expression level analysis of sequenced small RNAs in the three developmental phases in *N. lugens*.

Figure S3. Selected KEGG pathways derived from candidate target genes of DEMs (10 of 197 enriched KEGG pathways) of *N. lugens*. The *x-axis* represents the number of target genes. The y-*axis* represents the predicting involvement in reproduction KEGG pathways.

Figure S4. Gene Ontology (GO) annotation and classification of predicated target genes corresponding to 43 DEMs in *N. lugens*. The results are classified into three groups: biological process, cellular component and molecular function. The *x-axis* represents the different terms. The *y-axis* represents the number of genes.

Figure S5. The effect of the injection of miRNA mimics on fecundity in *N. lugens*. Newly emerged SW female adults were injected with miR-9, miR-275 and miR-317 mimics, and mimic-NC was injected as a negative control. Results are the mean±SEM of 20 independent experiments.

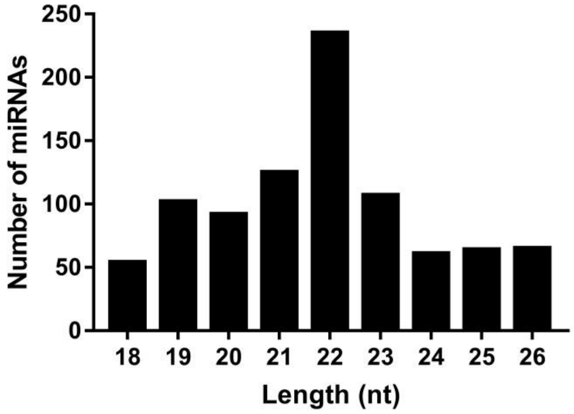

Figure S1. Length distribution of identified small RNAs in the three developmental phases in *N. lugens*.

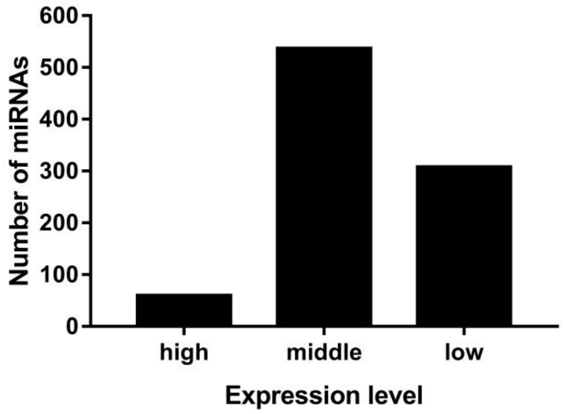

Figure S2. Expression level analysis of sequenced small RNAs in the three developmental phases in *N. lugens*.

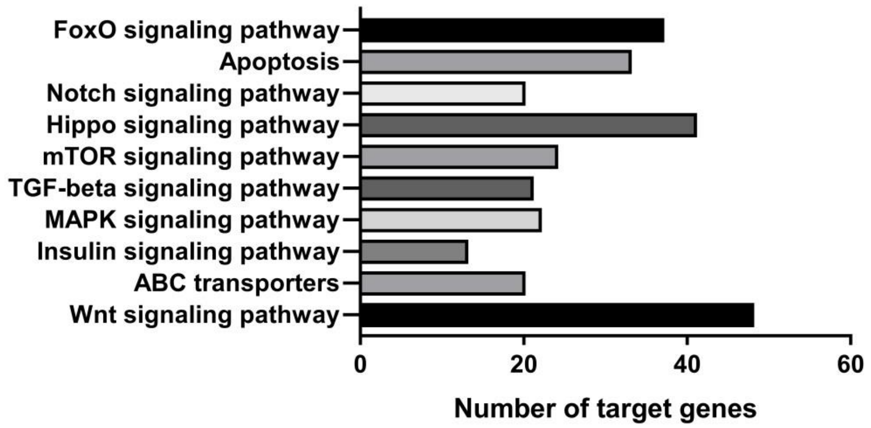

Figure S3. Selected KEGG pathways derived from candidate target genes of DEMs (10 of 197 enriched KEGG pathways) of *N. lugens*. The *x-axis* represents the number of target genes. The y-*axis* represents the predicting involvement in reproduction KEGG pathways.

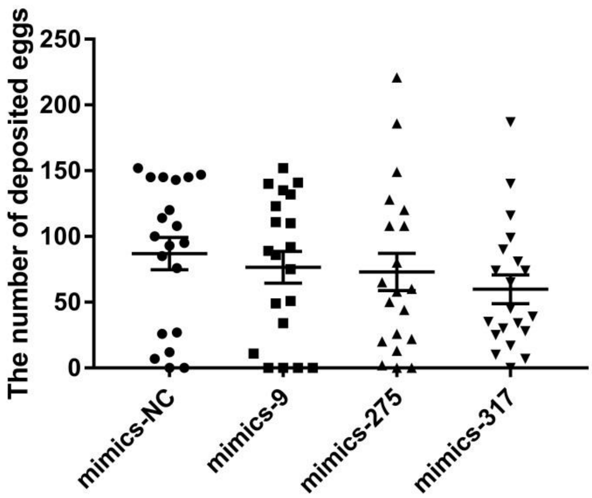

Figure S4. The effect of the injection of miRNA mimics on fecundity in *N. lugens*. Newly emerged SW female adults were injected with miR-9, miR-275 and miR-317 mimics, and mimic-NC was injected as a negative control. Results are the mean±SEM of 20 independent experiments.

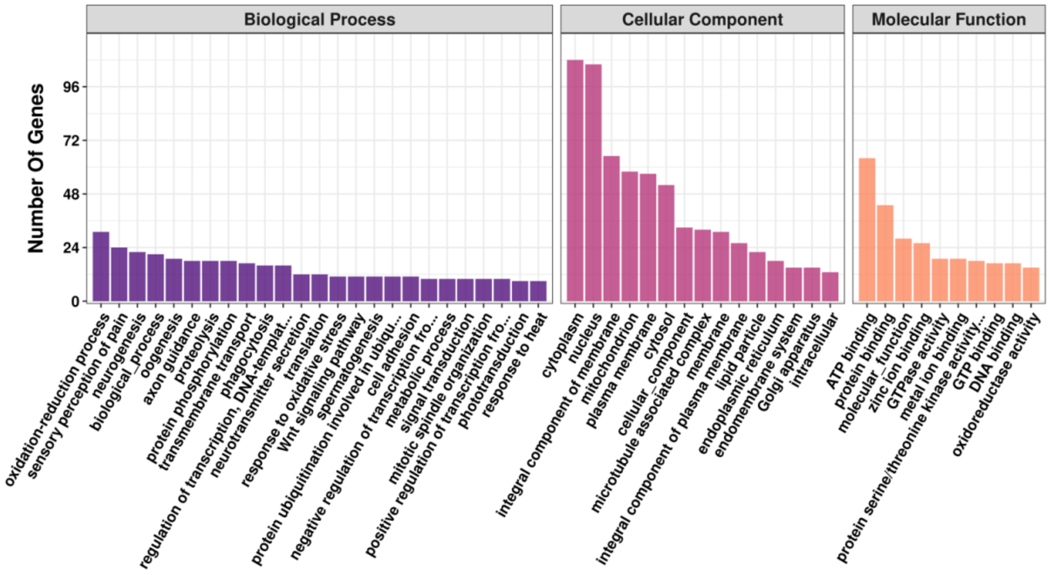

Figure S5. Gene Ontology (GO) annotation and classification of predicated target genes corresponding to 43 DEMs in *N. lugens*. The results are classified into three groups: biological process, cellular component and molecular function. The *x-axis* represents the different terms. The *y-axis* represents the number of genes.
